# FOLFOX Induced Takotsubo Cardiomyopathy Treated with Impella Assist Device

**DOI:** 10.1155/2017/8507096

**Published:** 2017-03-07

**Authors:** Swethika Sundaravel, Abdulah Alrifai, Mohamad Kabach, Waqas Ghumman

**Affiliations:** ^1^Internal Medicine, University of Miami, Palm Beach Regional Consortium, Atlantis, FL, USA; ^2^Cardiology, University of Miami, Palm Beach Regional Consortium, Atlantis, FL, USA

## Abstract

Chemotherapy induced cardiotoxicity is becoming increasingly prevalent with several new agents being used recently. The incidence of Takotsubo cardiomyopathy due to 5-fluorouracil based chemotherapeutic regimens like FOLFOX is not uncommon. It is also seen with platinum based chemotherapy. Most of these patients have reversible cardiotoxicity and the cardiac function recovers within a short period with supportive treatment. Here we have a patient who presented with cardiogenic shock after 5 days of receiving FOLFOX regimen for colorectal adenocarcinoma. She was treated with a percutaneous left ventricular assist device, Impella CP, for hemodynamic support with excellent outcome.

## 1. Case Description

This is a 61-year-old woman with history of paroxysmal atrial flutter status afrer ablation who was recently diagnosed with poorly differentiated adenocarcinoma of colon status after hemicolectomy. She had no other past medical history and was not taking any medications. She was a nonsmoker and nonalcoholic and denied use of illicit drugs. She was started on adjuvant chemotherapy with FOLFOX (5FU + folinic acid + oxaliplatin). Patient was tolerating the regimen without any complications until day 5 when she started experiencing acute dyspnea and diaphoresis. She was rushed to the emergency department. She was found to be hypotensive and in profound respiratory distress from pulmonary edema with extensive crackles bilaterally on chest auscultation. She was tachypneic and her oxygen saturation was 72% with 5 liters of nasal oxygen. She had to intubated immediately.

**Figure 1:**
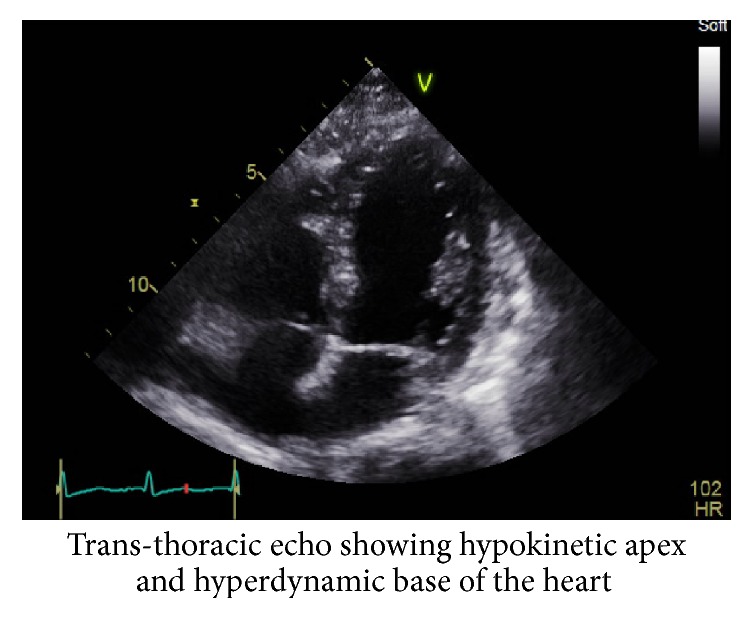
4-chamber view transthoracic echocardiogram.

Her ECG showed T inversions in the precordial leads and ST flattening in the inferolateral leads. Troponin was 0.41 ng/mL and trended up to 1.7 ng/mL in 12 hrs. NT-pro-BNP was 7392 pg/mL. Chest X-ray revealed bilateral pulmonary edema. Bedside 2D transthoracic echocardiogram (Figure 1) was done which showed hyperdynamic contraction of the basal region and stunning of the apex with a left ventricular ejection fraction of 25–30% (echo 1 month ago, before starting chemotherapy had normal ejection fraction).

**Figure 2:**
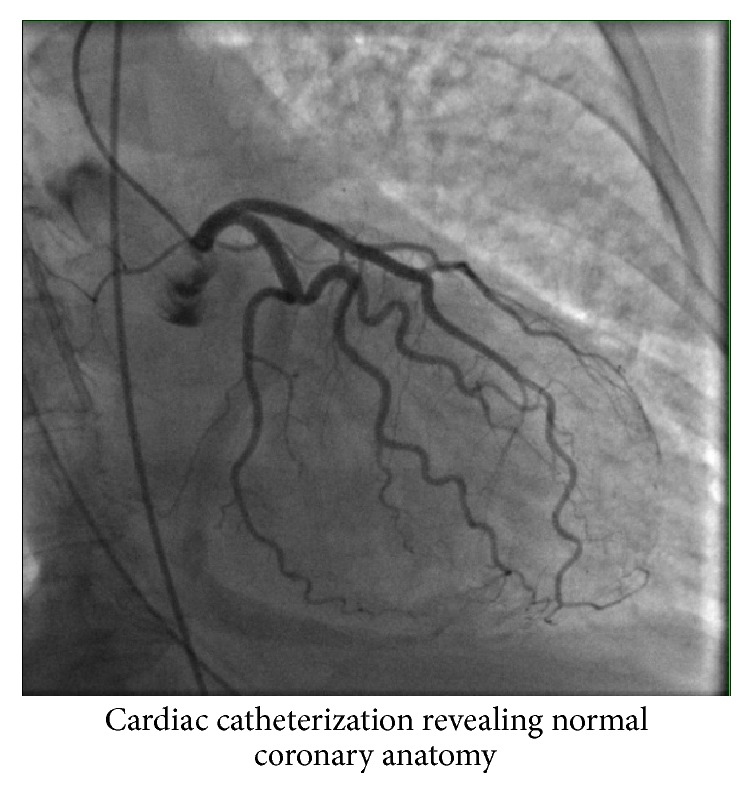
Left coronary artery.

**Figure 3:**
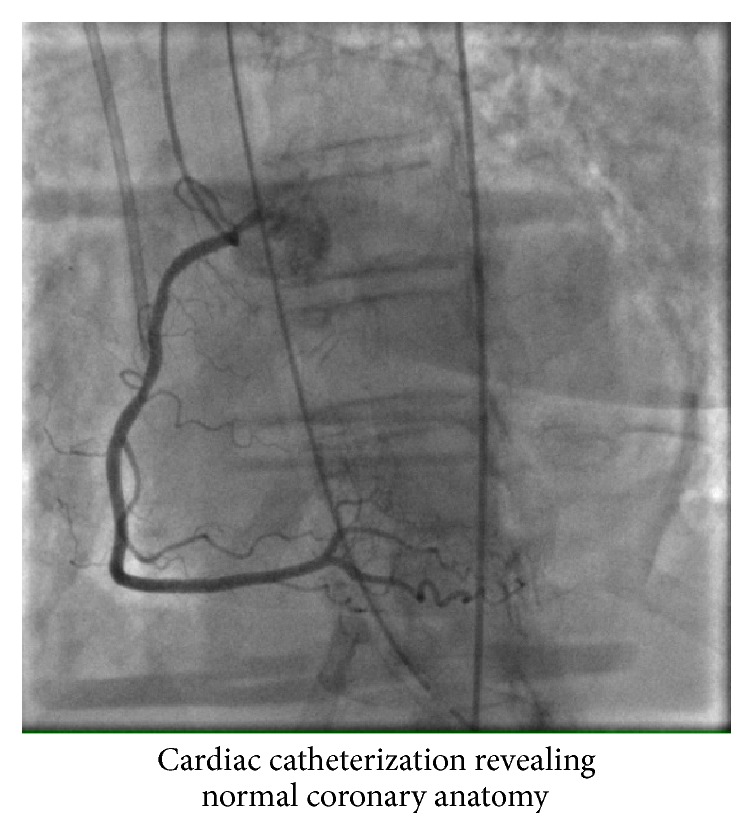
Right coronary artery.

**Figure 4:**
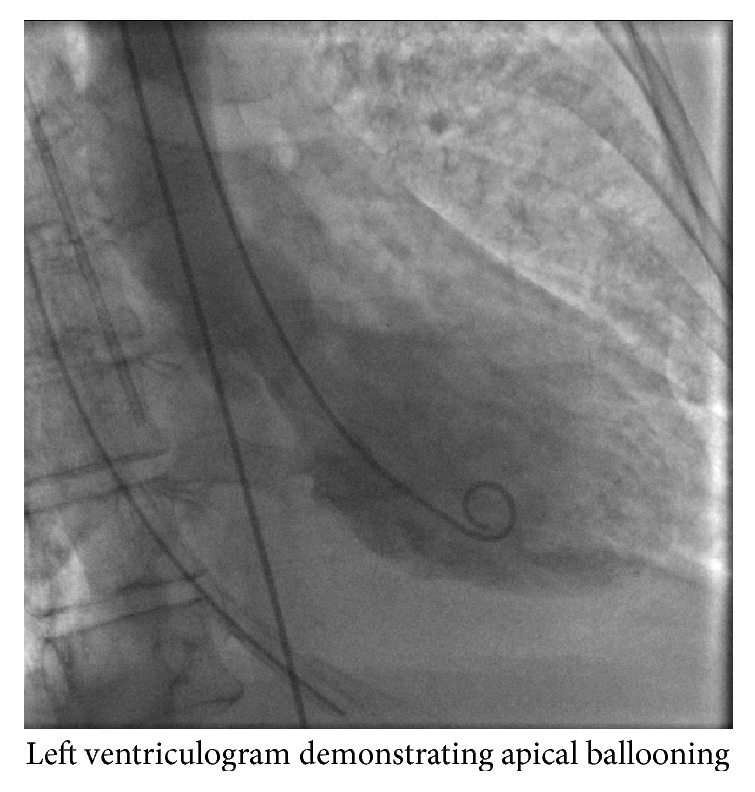
Left ventriculogram.

She underwent cardiac catheterization (Figures 2 and 3) which revealed normal flow in all the coronary arteries and left ventriculogram ejection fraction of 20% (Figure 4). Left Ventricular End Diastolic Pressure (LVEDP) was 25 mmHg and mean arterial pressure was less than 50 mmHg. Right heart catheterization demonstrated Pulmonary Capillary Wedge Pressure (PCWP) of 22 mmHg with mean pulmonary artery pressure of 29 mmHg. Her initial cardiac index was 1.5–2.1 liters/min/m^2^ and the cardiac output was 2.5–3 liters/min. Lactic acid level in the blood was 6 mmol/liter. An Impella CP assist device was placed for cardiac support. She also had a Swan-Ganz catheter in place for close hemodynamic monitoring. She also received diuresis and respiratory support. Over the next 3 days, she recovered remarkably. Her cardiac index improved to 2.5–3 liters/min/m^2^ and the mean pulmonary artery pressure improved to 19 mmHg. The lactic acid in the blood also came down to 1.5 mmol/liter. Echocardiogram revealed an ejection fraction of 35–40% with some contractile activity of the apical segments as well. The device was removed on day 3. She was also slowly weaned off pressor support and extubated in the subsequent days. She was discharged on day 7 with beta-blockers, expected to follow up with oncology for a different regimen.

## 2. Discussion

Takotsubo cardiomyopathy is defined as nonischemic cardiomyopathy which occurs usually due to stress and increased catecholamine levels in the blood. Also known as apical ballooning syndrome, it is associated with a transient left ventricular hypokinesis with ECG changes and troponin elevation in the setting of nonobstructive coronary arteries and absence of pheochromocytoma or other usual causes of myocarditis [1]. It was first reported in Japan, in 1990 [2]. It is most prevalent in women over the age of 50 years, although it is not uncommon in men. 4 types of Takotsubo cardiomyopathy have been described so far: apical, mid-ventricular, basal, and focal hypokinesis [3]. The incidence of Takotsubo cardiomyopathy after 5FU based chemotherapy is becoming increasingly prevalent [4]. Several cases have been reported in the literature [5, 6].

The pathogenesis of this condition is believed to be due to coronary vasospasm mediated 5FU and its metabolites [7]. Other possible mechanisms are direct cardiotoxicity of 5FU on the myocytes and endothelial damage. The incidence of cardiotoxicity is less with bolus regimens [8] than with continuous infusions or with coadministration of leucovorin [9] or cisplatin [10]. The usual clinical presentation is angina-like chest pain during the infusion of the drug with concomitant ECG changes [11]. It can also present with arrhythmias and cardiac arrest in rare instances [12]. About 2% of cases present in cardiogenic shock, like our patient [13]. Almost 95% of patients recover to normal cardiac function, but sometimes mortality rates can be as high as 8% [14]. Our patient was quite different in presentation from the rest. She presented on day 5 after the chemotherapy with cardiogenic shock.

The treatment is usually supportive with nitrates and calcium channel blockers in the acute phase along with hemodynamic support in the form of ventricular assist devices or Extracorporeal Membrane Oxygenation (ECMO) if needed. These patients must be started on beta-blockers for long-term treatment. In chemotherapy induced cases, discontinuing the offending agent is almost always inevitable. Following resolution of the acute cardiotoxic phase, reinstitution of the same agent has proven to be fatal in some cases [15] and recurrence of Takotsubo has also been reported [16]. Considering these life-threatening complications, it is not advisable to rechallenge the patient with the same chemotherapeutic agent [17].

The use of cardiac assist devices for acute cardiogenic shock is well known. There are several types of devices including Intra-Aortic Balloon Pumps (IABP), Impella devices, tandem heart, and Left Ventricular Assist Devices (LVAD). The use of Impella device for acute cardiogenic shock is becoming more popular because of its immediate augmentation of the left ventricular function and its ability to supplement the cardiac output to about 2.5–5 liters/min. It is used regularly for acute coronary syndromes and nonischemic cardiomyopathies including Takotsubo cardiomyopathy [18, 19]. It is proven to be more effective, feasible, and safe compared to Intra-Aortic Balloon Pumps by providing superior hemodynamic support [20]. To our knowledge and on review of literature, there is no reported case of FOLFOX or chemotherapy induced Takotsubo cardiomyopathy that has been treated with Impella device. Our patient had severe cardiogenic shock with an EF of 20% which necessitated the Impella assist device. We used Impella CP which provided an additional cardiac output of about 2.5–3 liters/min. The patient's cardiac function improved remarkably in 3 days and the device was removed. There were no complications encountered.

## 3. Conclusion

5-Fluorouracil based chemotherapeutic regimens are extremely potent in treating several cancers including high grade colorectal malignancies. The most important and most dangerous side-effect of this drug is the cardiotoxicity which can manifest in many ways ranging from subtle angina to florid cardiogenic shock and cardiac arrest from ventricular fibrillation. Several other chemotherapeutic agents like platinum based therapies are also associated with cardiotoxicity. Cardiac oncology is a relatively new field of cardiology dealing with cancer patients who have suffered cardiotoxicity from chemotherapy. Several novel strategies have been tested to minimize the side-effects of these drugs including prophylactic nitrates, calcium channel blockers, and ACE inhibitors/ARBs to these patients, but, so far, none of them have been proven effective except dose reduction of the chemotherapeutic agent and bolus regimens.

The treatment of acute cardiogenic shock has also come a long way with the discovery of new percutaneous ventricular assist devices which have proven to be very effective in the decompensated setting. The use of these devices in chemotherapy induced cardiomyopathy has shown to decrease the number of hospital/ICU days and result in speedy recovery. More randomized trials and systematic reviews are required to analyze the overall use of such devices.
